# Divergent bufavirus harboured in megabats represents a new lineage of parvoviruses

**DOI:** 10.1038/srep24257

**Published:** 2016-04-26

**Authors:** Michihito Sasaki, Gabriel Gonzalez, Yuji Wada, Agus Setiyono, Ekowati Handharyani, Ibenu Rahmadani, Siswatiana Taha, Sri Adiani, Munira Latief, Zainal Abidin Kholilullah, Mawar Subangkit, Shintaro Kobayashi, Ichiro Nakamura, Takashi Kimura, Yasuko Orba, Kimihito Ito, Hirofumi Sawa

**Affiliations:** 1 Division of Molecular Pathobiology, Research Center for Zoonosis Control, Hokkaido University, Sapporo, Japan; 2 Division of Bioinformatics, Research Center for Zoonosis Control, Hokkaido University, Sapporo, Japan; 3 Laboratory of Veterinary Pathology, Faculty of Veterinary Medicine, Bogor Agricultural University, Bogor, Indonesia; 4 Veterinary Investigation and Diagnostic Center, Bukittinggi, Indonesia; 5 Faculty of Agriculture, Gorontalo State University, Gorontalo, Indonesia; 6 Faculty of Animal Husbandry, Sam Ratulangi University, Manado, Indonesia; 7 Office of Animal Husbandry and Fisheries, Soppeng, Indonesia; 8 Veterinary Medicine Study Program, Faculty of Medicine, Hasanudin University, Makassar, Indonesia; 9 Unit of International Cooperation, Research Center for Zoonosis Control, Hokkaido University, Sapporo, Hokkaido, Japan; 10 Global Institution for Collaborative Research and Education, Hokkaido University, Sapporo, Hokkaido, Japan; 11 Global Virus Network, Baltimore, MD, USA

## Abstract

Bufavirus is a recently recognized member of the genus *Protoparvovirus* in the subfamily *Parvovirinae*. It has been reported that human bufavirus was detected predominantly in patients with diarrhoea in several countries. However, little is known about bufavirus or its close relatives in nonhuman mammals. In this study, we performed nested-PCR screening and identified bufavirus from 12 megabats of *Pteropus* spp. in Indonesia. Furthermore, we determined nearly the full genome sequence of a novel megabat-borne bufavirus, tentatively named megabat bufavirus 1. Phylogenetic analyses showed that megabat bufavirus 1 clustered with known protoparvoviruses, including human bufavirus but represented a distinct lineage of bufavirus. Our analyses also inferred phylogenetic relationships among animal-borne bufaviruses recently reported by other studies. Recombination analyses suggested that the most common recent ancestor of megabat bufavirus 1 might have arisen from multiple genetic recombination events. These results characterized megabat bufavirus 1 as the first protoparvovirus discovered from megabats and indicates the high genetic divergence of bufavirus.

Bufavirus (BuV) is a recently recognized parvovirus and was initially identified in the faeces of a child with diarrhoea in Burkina Faso in 2012^1^. Then, multiple studies described BuV in the faeces of humans in Tunisia, Finland, the Netherlands, Bhutan, Thailand, China and Turkey^1^,^2^,^3^,^4^,^5^,^6^,^7^, indicating its worldwide distribution. BuV was detected predominantly in human patients with diarrhoea or gastroenteritis, while the pathogenicity of BuV remains to be elucidated because of the failure to recover infectious BuV from samples. All human-borne BuVs are phylogenetically distinct from other known members of the subfamily *Parvovirinae* and now belong to a new species, primate protoparvovirus 1 of the genus *Protoparvovirus* in the subfamily *Parvovirinae*^8^.

Previous viral metagenomic studies identified close relatives of human-borne BuV in animals. In an earlier study, we described distinct lineages of BuV in wild shrews and baboons in Zambia^9^. Other studies also reported different BuVs or related parvoviruses in laboratory rhesus monkeys in the USA^10^, wild rats in China^11^, domestic pigs in Hungary^12^ and in wild microbats in Hungary and China^13^,^14^. Although these previous studies have demonstrated the presence of animal-borne BuVs, to date there has been no phylogenetic analysis that has covered all published bufaviruses and related protoparvoviruses. Therefore, the phylogenetic relationships among these viruses are still poorly understood.

Bats are traditionally divided into two suborders: *Megachiroptera* (megabats) and *Microchiroptera* (microbats). Megabats and microbats mainly consist of frugivorous bats and insectivorous bats, respectively^15^. In addition to their biological differences, they are phylogenetically distinguishable^16^,^17^. Megabats are a potential reservoir of emerging zoonotic viruses, including Nipah virus, Hendra virus, SARS coronavirus and Ebola virus^18^,^19^. In addition, a metagenomic study showed that megabats harbour various mammalian viruses, some of which are genetically related to human viruses^20^. However, BuVs or related parvoviruses were detected in microbats but not in megabats. In the present study, we investigated BuVs in flying foxes (*Pteropus* spp.), which belong to the megabat family. Based on the obtained sequence data, we examined the phylogenetic relationships between the identified-BuVs and previously identified BuVs. One of the BuVs identified in this study was further characterized and we obtained a nearly full genome sequence.

## Results

A total of 183 megabats consisting of four different species were included in this study (Table 1). Among these, *Pteropus* sp. and *Dobsonia* sp. were not identified at species level, but other genetically close species *Pteropus hypomelanus* and *Dobsonia moluccensis*, were included^21^. In an earlier study, BuV was detected from the spleen tissues and faeces of shrews^9^. Therefore, we used nested-PCR targeting for the *NS1* gene of BuV using 183 spleens and 96 faeces samples (faecal samples were unavailable for a subset of megabats). As shown in Table 1, BuV was identified in three spleen and nine faeces specimens from *Pteropus vampyrus*, and one spleen specimen from *Pteropus* sp. Among these, one *Pteropus vampyrus* in Magelang was positive for BuV in both the spleen and faeces. With a limited number of samples, the detection rate of BuV in spleen (2%) was lower than that in faeces (9%).

Based on the nucleotide sequence identity, we tentatively grouped and named the identified BuVs into three strains: megabat bufavirus 1 (MgBuV1) originating from *Pteropus vampyrus* in Surabaya and Magelang (accession numbers LC085668 to LC085676), megabat bufavirus 2 (MgBuV2) from *Pteropus vampyrus* in Lima Puluh Kota (LC085665 and LC085666), and megabat bufavirus 3 (MgBuV3) from *Pteropus* sp. in Paguyaman (LC085667). The partial *NS1* gene fragment of MgBuV1 showed 81–83% and 91–92% nucleotide identity to MgBuV2 and MgBuV3, respectively. The corresponding region of MgBuV2 was 80% identical to that of MgBuV3.

**Figure 1:**
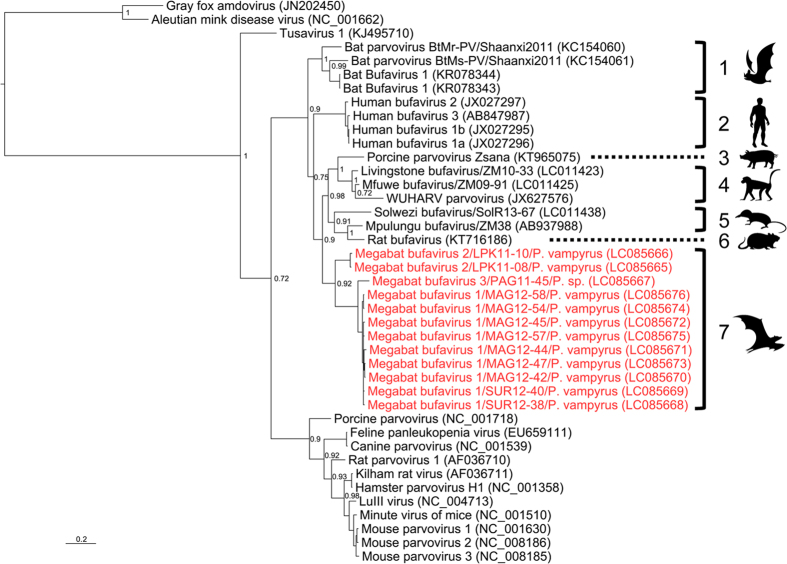
Phylogenetic analysis based on the amplicon sequences and reference sequences of the *NS1* gene from protoparvoviruses. The analysed region corresponds to nucleotide positions 946 to 1379 in the human bufavirus 1a genome (GenBank accession number JX027296). The sequences identified in this study are coloured in red. The respective GenBank accession numbers of the viral sequences are shown in parentheses. Bayesian posterior probabilities are indicated at major nodes. The scale bar represents a distance of 0.2 substitutions per site. Images were provided by Ms. E. Hayashi with permission.

Currently, DDBJ/EMBL/GenBank databases contain complete or partial nucleotide sequences of BuV from humans, nonhuman primates, shrews, rats, pigs and microbats. A phylogenetic tree was generated on the basis of the identified sequences of *NS1* gene in this study and a corresponding region of the available reference sequences of protoparvoviruses including BuVs (Fig. 1). Known BuVs formed subgroups by host were distinguishable from other protoparvoviruses (groups 1–6, Fig. 1). MgBuV1, MgBuV2 and MgBuV3 diverged from a common ancestor of known protoparvoviruses and were likely to form a distinct subgroup from other BuVs, including microbat-borne BuVs (group 7, Fig. 1).

**Figure 2:**
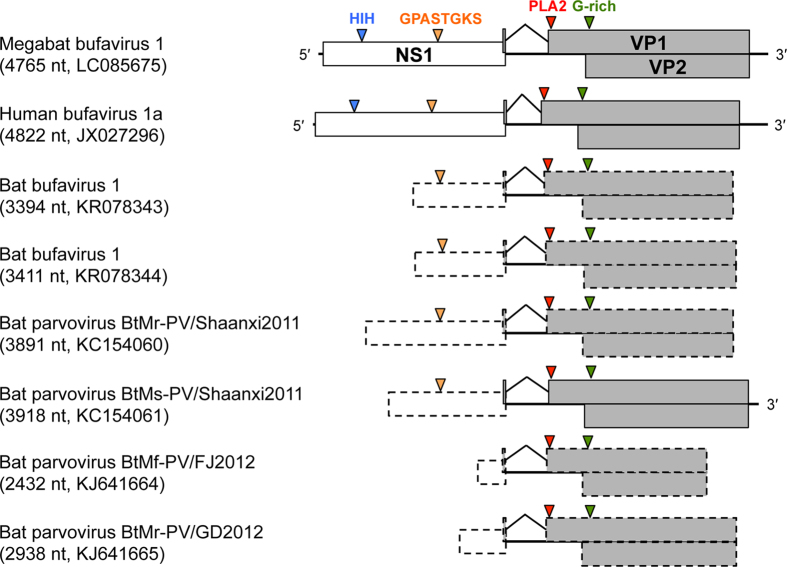
Genome organization of megabat bufavirus 1 and microbat-borne bufaviruses reported previously. The length of the determined nucleotide sequences and GenBank accession numbers of the viral sequences are shown in parentheses. Solid-lined or dashed-lined boxes indicate complete or incomplete sequenced ORFs, respectively. Blue, orange, red and green arrowheads on NS1 and VP1 indicate the position of conserved amino acid motifs of parvoviruses.

To reveal the whole genome sequence and genetically characterise further the identified BuVs, we subjected DNA samples isolated from spleens and faeces to next-generation sequencing. A small number of sequence reads from MgBuV1, but not MgBuV2 and MgBuV3, enabled to amplify the overlapping large fragments by PCR and determine nearly the whole genome sequence, including the complete protein-coding region. The obtained genome sequence of MgBuV1 (accession number LC085675) was 4765 nt in size and encoded three complete ORFs: the non-structural protein (NS1) and the viral capsid proteins (VP1 and VP2). These proteins showed 46–61% amino acid sequence identities to those of other BuVs of which complete genome sequences were available (Table 2). Parvovirus-conserved amino acid motifs were found in each viral protein of MgBuV1 (Fig. 2). NS1 contained a putative endonuclease metal coordination motif “HIH” and the helicase motif Walker A “GPASTGKS”. The VP1 of most parvovirus species possesses a phospholipase A2 (PLA2) motif that is required for viral entry^22^. Three potential sites responsible for the enzymatic activity of PLA2 were strictly conserved in the VP1 of MgBuV1: a calcium-binding loop site “YLGPG” and two catalytic sites “HDLEY” and “D”. The N-terminus of the VP1 and VP2 of MgBuV1 had a glycine-rich motif (G-rich) required for the cellular entry of parvovirus^23^.

**Figure 3:**
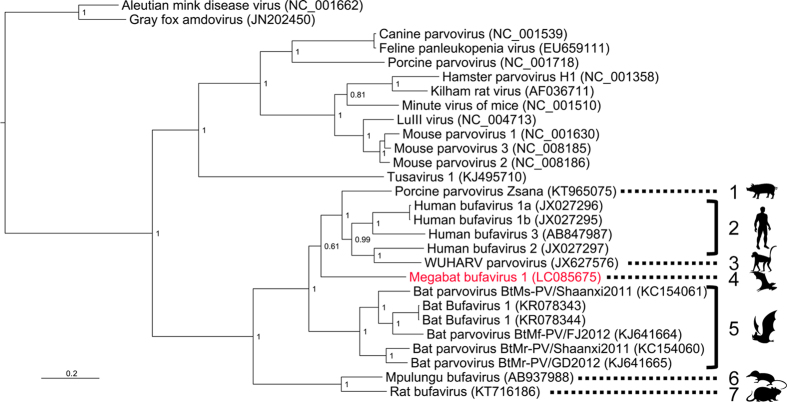
Phylogenetic analysis based on the *VP2* gene. The sequence identified in this study is coloured in red. The respective GenBank accession numbers of the viral sequences are shown in parentheses. Bayesian posterior probabilities are indicated at each node. The scale bar represents a distance of 0.2 substitutions per site. Images were provided by Ms. E. Hayashi with permission.

Recently, six partial genome sequences of human BuV-like parvovirus were identified from insectivorous microbats in Hungary and China^13^,^14^. These sequences covered a partial coding region of *NS1* and almost complete to complete coding regions of *VP1* and *VP2* (Fig. 2). Therefore, using the coding region of *VP2*, we performed a phylogenetic analysis to infer the deeper phylogenetic relationships between these microbat-borne BuVs and megabat-borne MgBuV1 (Fig. 3). All microbat-borne BuVs formed a single cluster (group 5, Fig. 3). MgBuV1 branched from the common ancestor of human BuVs, non human primate-borne BuV (WUHARV parvovirus) and pig-borne BuV, and represented a distinct lineage of BuV (linage 4, Fig. 3). WUHARV parvovirus (linage 3, Fig. 3) fell into the cluster of human BuVs (group 2, Fig. 3), consistent with a previous study of past recombination events behind the origin of WUHARV parvovirus^13^.

**Figure 4:**
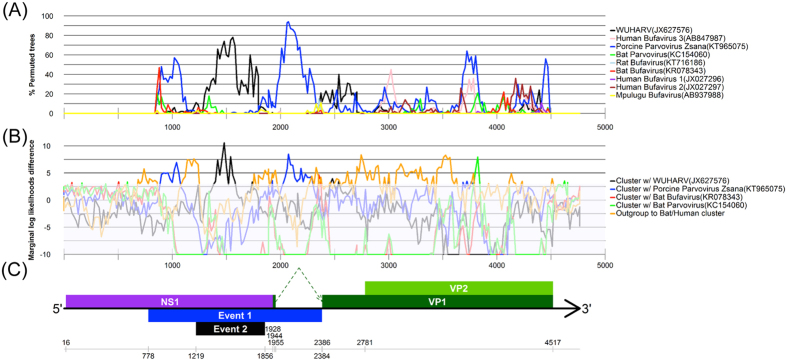
Recombination analyses of megabat bufavirus 1. Abscissae represent positions adjusted to reflect the position in the megabat bufavirus 1 genome. (**A**) Bootscan results supporting the clustering of megabat bufavirus 1 with other strains, which are color-coded as shown to the right. The ordinate shows the percentage of permuted trees supporting the clustering in each window. (**B**) Bayesian marginal likelihood support for the topological position of megabat bufavirus 1. The ordinate shows the log difference for the marginal likelihood of the model assuming the topological hypothesis and the model assuming the opposite. Lines below the threshold support (<3) are shown as a lighter colour. (**C**) Genome representation showing the supported recombination events and the position relative to the protein encoding regions. Event 1 and Event 2 show the supported recombination events between MgBuV1 and porcine parvovirus Zsana or between MgBuV1 and WUHARV parvovirus, respectively. Nucleotide positions 778, 1219, 1856 and 2384 show putative recombination breakpoints.

**Figure 5:**
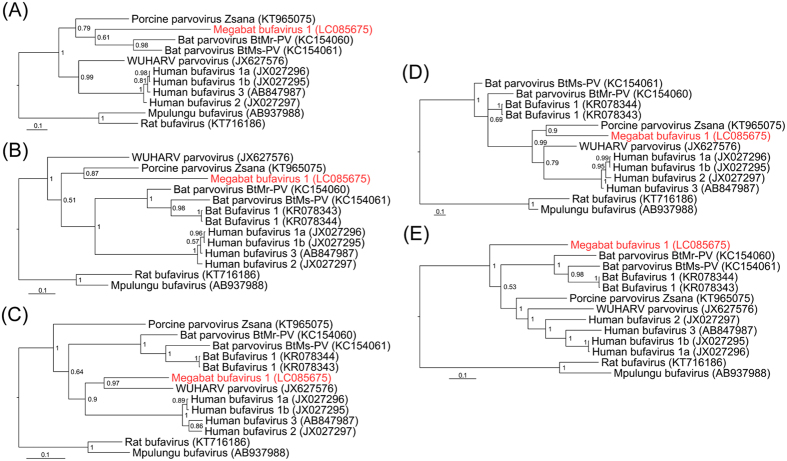
Separate phylogenetic analyses of five regions bordered by putative recombination breakpoints. Phylogenetic trees were inferred from nucleotide positions 1–778 (**A**) 778–1219 (**B**) 1219–1856 (**C**) 1856–2384 (**D**) 2384–4765 (**E**) on the megabat bufavirus 1 genome. The respective GenBank accession numbers of the viral sequences are shown in parentheses. Bayesian posterior probabilities are indicated at each node. The scale bars represent a distance of 0.2 substitutions per site.

We searched for putative recombination events on the genome of MgBuV1 using two different approaches based on sliding window analyses, one using the Bootscan approach and the second using Bayesian inference (Fig. 4A,B). Both analyses suggested a mosaic genome structure in MgBuV1 and possible recombinations among ancestors of MgBuV1, WUHARV parvovirus and porcine parvovirus Zsana, around nucleotide positions 778 to 2384 of the MgBuV1 genome (Fig. 4C). These observations were supported by independent phylogenetic trees built from five regions bordered by the inferred recombination breakpoints (Fig. 5A–E). Among the BuVs and related protoparvoviruses, MgBuV1 branched from the ancestor of porcine parvovirus Zsana in the phylogenetic tree at position 778 to 1219 (Fig. 5B) and position 1856 to 2384 (Fig. 5D). However, MgBuV1 branched from its ancestor WUHARV parvovirus at position 1219 to 1856 in the phylogenetic tree (Fig. 5C). In contrast, MgBuV1 formed a distinct lineage in phylogenetic trees constructed from position 2384 to 4765 (Fig. 5E). Based on this evidence, we inferred the parsimonious origin of the mosaic genome in MgBuV1 from both recombination analyses (Fig. 4C).

## Discussion

Since the discovery of human BuV in 2012, its genetic divergence and worldwide distribution have been updated by a series of reports^1^,^2^,^3^,^4^,^5^,^6^,^7^. Although recent studies described BuVs in nonhuman mammals, little is known about the epidemiology of animal-borne BuVs.^9^,^10^,^11^,^12^,^13^,^14^. In this study, we report for the first time, a BuV identified in megabats. MgBuV1 is the fourth animal borne-BuV where the whole genome sequence of the protein-coding region has been determined. In addition, the detection of partial NS1 gene sequences different from MgBuV1 (called MgBuV2 and MgBuV3) indicates the presence of MgBuV1 variants. The discovery of megabat-borne BuVs facilitates a better understanding of the diversity and host-range of BuV.

MgBuV1 has a similar genome organization and is phylogenetically related to other mammalian-borne BuVs. According to the species demarcation criteria of the International Committee on Taxonomy of Viruses, parvoviruses in the same species should share >85% amino acid sequence identity in their whole NS1^8^. As shown in Table 2, the NS1 of MgBuV1 showed <60% identity to those of other related parvoviruses. Taken together, these results suggest that MgBuV1 is a novel species of protoparvovirus harboured by megabats.

Recently, complete or partial genome sequences of BuVs related to human BuV were identified from nonhuman primates, shrews, rats, pigs and microbats^9^,^10^,^11^,^12^,^13^,^14^. However, the phylogenetic relationships among these viruses remains to be elucidated. Our phylogenetic analyses covered the available sequences of these BuVs and showed a large degree of genetic divergence of BuV in the genus *Protoparvovirus*. Both phylogenetic analyses based on NS1 and VP2 genes showed that a group of BuVs comprised host-specific lineages and megabat-borne BuVs were clearly distinguished from microbat-borne BuVs. While the phylogeny of BuV is likely to be associated with their host, further studies of BuVs in animals are needed to infer the virus-host coevolution.

Genome recombination is a compelling argument to explain the evolution of a variety of viruses^24^,^25^; furthermore, evidence for recombination events have been also observed in the genome of some protoparvoviruses^13^,^26^,^27^,^28^. In this study, we revealed putative recombined regions and phylogenetic incongruences across the MgBuV1 genome, suggesting genetic recombination between MgBuV1 and porcine parvovirus Zsana (event 1, Fig. 4C) and other recombination events between MgBuV1 and WUHARV parvovirus (event 2, Fig. 4C). These results imply that the most common recent ancestor of MgBuV1 might have arisen from multiple recombination events. Such events also imply the occurrence of dual infection with ancestors of MgBuV1, WUHARV parvovirus and porcine parvovirus Zsana in nature.

In conclusion, we demonstrated the presence of megabat-borne BuV, the first protoparvovirus discovered from megabats. Further genetic characterization revealed the phylogenetic distinctiveness of MgBuV1 and suggested a molecular evolution of MgBuV1 driven by multiple recombination events. These results provide new insights into the high genetic divergence of BuV and will help us to investigate further BuV in animals.

## Methods

### Ethics statement and sample collection

All experiments involving animals were performed in accordance with the ethical guidelines of the Animal Care and Use Committee of Veterinary Teaching Hospital, Bogor Agricultural University. The protocol was approved by the Animal Care and Use Committee of Veterinary Teaching Hospital, Bogor Agricultural University (permit number 05-2010 RSHP-IPB). Collection and exportation of samples from megabats were performed with the permission of the Directorate General of Livestock and Animal Health Services, Ministry of Agriculture, Republic of Indonesia. Faecal and spleen samples were collected from megabats at eight regions in Indonesia from 2010 to 2014. Some samples were used for different research projects, as reported previously^21^,^29^,^30^,^31^. Species were identified based on the morphological features and nucleotide sequence analysis of both mitochondrial 16S rRNA and cytochrome *b* as described previously^21^. Sample information is summarized in Table 1.

### Nested-PCR screening

Faeces suspended in RNAlater reagent (Ambion, Life Technologies, Carlsbad, CA) were subjected to DNA extraction using a High Pure Viral Nucleic Acid kit (Roche Diagnostics GmbH, Mannheim, Germany) or QIAamp Viral RNA Mini Kit (Qiagen, Valencia CA). DNA from spleen tissues was prepared using DNAzol reagent (Molecular Research Center, Cincinnati, OH) or QIAamp DNA Mini kit (Qiagen). For nested PCR screen targeting for *NS1* gene of BuV, we followed the method of Sasaki *et al.*^9^.

### Complete genome sequencing

To determine the complete genome sequence of megabat BuV, a DNA library was prepared from the faecal-DNA samples using the Nextera XT DNA sample preparation kit (Illmina, San Diego, CA) and were sequenced on an illumina MiSeq instrument using the MiSeq Reagent Kit v3 600-cycle (Illumina) according to the manufacturer’s instructions. The sequence reads homologous to known BuVs were identified by read mapping using CLC Genomics Workbench (CLC bio, Aarhus, Denmark). The overlapping large fragments were amplified by conventional PCR with specific primer sets designed from each identified sequence read. The 3′ end of virus genome was amplified by the modified rapid amplification of cDNA ends as reported previously^32^. All the amplified fragments were assembled manually into a contiguous sequence.

### Phylogenetic analyses

Sequences were aligned with MAFFT^33^ using the FFT-NS-I method. Phylogenetic inferences were performed on multiple sequence alignments with MrBayes 3.2^34^ using a General Time Reversible model with a rate variation between sites modelled with a gamma distribution (+Γ) with six categories and allowing for a percentage of invariable sites (+I).

### Recombination analyses

A recombination analysis was performed in Simplot 3.5.1^35^ using the Bootscan algorithm with 200 nt window and 20 nt step under a Kimura (2-parameter) model^36^ and a Neighbor-Joining approach with 1000 repetitions.

Additionally, this study implemented a second analysis approach over the same sliding window that incorporated the power of Bayesian inference to support or reject hypotheses regarding the topology of the phylogenetic tree. For each window, a hypothesis of the clustering of two particular sequences was tested against the null hypothesis that the cluster was less likely than those sequences independently clustering with any other under the same model parameters. To maximize the inferential power of the approach, the marginal likelihoods of the models assuming opposite topological hypotheses were compared with the stepping stone^37^ sampling approach implemented in MrBayes 3.2. Then, the difference of the calculated marginal likelihoods for both models, i.e., the model assuming a clustering and the model without the clustering, represented the Bayes factor, which usually is interpreted as supporting the first model if the difference exceeds three and strongly supportive when it exceeds five^38^. All hypotheses were tested under the same model assumed for the phylogenetic analyses.

The dataset in both analyses was composed of sequences in the BuV cluster. Adopting a parsimonious approach, this study accepted only those events suggested by Bootscan that also had support from the Bayesian hypotheses testing.

### Nucleotide sequences

All the nucleotide sequences determined in this study were deposited in the DDBJ/EMBL/GenBank databases under accession numbers LC085665−LC085676.

## Additional Information

**How to cite this article**: Sasaki, M. *et al.* Divergent bufavirus harboured in megabats represents a new lineage of parvoviruses. *Sci. Rep.* **6**, 24257; doi: 10.1038/srep24257 (2016).

## Figures and Tables

**Table 1:** Sample information and nested-PCR screening results for bufaviruses.

Bat species	Location collected	Year collected	No. of samples PCR-positive/No. of samples tested	
			Spleen	Faeces
*Pteropus vampyrus*	Panjalu district	2010	0/26	NT***
	Lima Puluh Kota district	2011	2/20	NT
	Surabaya district	2012	0/3	2/3
	Magelang district	2012	1/20	7/19
*Pteropus* sp.*	Popayato district	2011	0/4	NT
	Paguyaman district	2011	1/23	NT
	Paguyaman district	2012	0/2	0/2
	Paguyaman district	2013	0/10	0/8
	Soppeng district	2014	0/7	0/7
	Sidrap district	2014	0/15	0/15
*Acerodon celebensis*	Paguyaman district	2012	0/18	0/18
	Paguyaman district	2013	0/18	0/7
*Dobsonia* sp.**	Paguyaman district	2012	0/17	0/17
Total			4/183	9/96

*Genetically closely related to *Pteropus hypomelanus*. **Genetically closely related to *Dobsonia moluccensis*. ***Not tested because of unavailability of faeces.

**Table 2:** ORF length and pairwise amino acid identities in NS1, VP1 and VP2 between Megabat bufavirus 1 and the indicated bufaviruses.

	Accession no.	Genome size(nt)	NS1		VP1		VP2	
			Length(aa)	Identity(%)	Length(aa)	Identity(%)	Length(aa)	Identity(%)
Megabat bufavirus 1	LC085675	4765	642	100	719	100	578	100
Human bufavirus 1	JQ918261	4912	671	46.0	707	59.3	569	57.5
Human bufavirus 2	JX027297	4562	673	52.6	707	61.4	569	60.0
Human bufavirus 3	AB847989	4766	673	53.5	710	61.4	572	59.7
WUHARV parvovirus	JX627576	4909	665	59.8	737	60.8	569	60.4
Mpulungu bufavirus	AB937988	4613	639	54.7	728	48.4	587	48.5
Rat bufavirus	KT716186	4634	644	54.5	726	48.7	585	48.4

nt, nucleotide; aa, amino acid; ORF, Open Reading Frame.
